# Comparison of ancient and modern Chinese based on complex weighted networks

**DOI:** 10.1371/journal.pone.0187854

**Published:** 2017-11-10

**Authors:** Xinru Cui, Jinxu Qi, Hao Tan, Feng Chen

**Affiliations:** 1 College of Electronic and Information Engineering, Southwest University, Chongqing, China; 2 School of Mathematics and Statistics, Southwest University, Chongqing, China; 3 Key Laboratory of Nonlinear Circuits and Intelligent Information Processing, Chongqing, China; 4 Chongqing Collaborative Innovation Center for Brain Science; IUMPA - Universitat Politecnica de Valencia, SPAIN

## Abstract

In this study, we compare statistical properties of ancient and modern Chinese within the framework of weighted complex networks. We examine two language networks based on different Chinese versions of the Records of the Grand Historian. The comparative results show that Zipf’s law holds and that both networks are scale-free and disassortative. The interactivity and connectivity of the two networks lead us to expect that the modern Chinese text would have more phrases than the ancient Chinese one. Furthermore, by considering some of the topological and weighted quantities, we find that expressions in ancient Chinese are briefer than in modern Chinese. These observations indicate that the two languages might have different linguistic mechanisms and combinatorial natures, which we attribute to the stylistic differences and evolution of written Chinese.

## 1 Introduction

With the publication of seminal works [1,2], complex networks have become a popular research topic in statistics, sociology, biology, and other fields over the past 20 years [3–6]. Investigation of real-world networks of various kinds has led to the study of many complex systems from the viewpoint of complex networks including ecology [7], computation [8], coding [9], cell and molecular biology [10], protein [11], neuroscience [12–14], human brain [15–17], and communication networks [18]. These studies indicate that understanding of the qualitative and quantitative characteristics of complex networks can be of great assistance in analyzing a variety of complex systems.

Naturally, a human language network can also act as a kind of complex adaptive system [19]. Chinese has been regarded as one of the most important languages in the world, with Chinese characters playing an important role in its well-known civilization [20,21]. In modern Chinese, a meaningful Chinese word consists of many characters, typically requiring artificial division because the classification criterion is restricted to historical evolution. In ancient Chinese, the implication of a single character is substantially different from that in modern Chinese, wherein it tends to have the function of a phrase. Moreover, even the grammar and structure of ancient Chinese are totally different compared to modern Chinese. As the basic unit of written Chinese, characters have a strict logical rationality of structure and have provided a deep-rooted concept in Chinese [22]. Based on the evolutionary process of Chinese, we find that these characters have been a stable factor throughout history. Characters are adopted for not only the analysis of the Chinese language structure evolving over time but also the exploration of the characteristics of language organization. Therefore, focusing on the structure of characters might help us to improve our understanding of Chinese.

Sentences in Chinese consist of characters and words in which the most correlated characters in a sentence are usually the closest [23]. Thus, Chinese language networks can be interpreted in terms of the adjacency relationship between characters appearing in the text. If we treat each character unit as a point and the relationship between the units as a line, then we can use a network to simulate the structure, function, and evolution of the language [24]. Furthermore, we can analyze the differences between two different language networks. Recently, some scholars have explored language networks in those terms, with many important linguistic properties being discovered by Li [20] and Liang [25]. However, they discussed only undirected and unweighted networks, and no comparisons have been made regarding the characteristics of different dialects or stages of Chinese. Therefore, in our research, the main purpose is to investigate the similarities and differences between the language networks for ancient (ALN) and modern (MLN) Chinese. Both networks are constructed in the same manner and treated as weighted directed graphs. Some articles suggest ways to connect graphs and network by graph entropy, graph distance measure and so on[26–31].

The remainder of this paper is organized as follows. In Section 2, we briefly introduce the construction of the two character networks, and in Section 3, we show the analytical results of the two networks, such as their degree distribution and clustering. Discussion and conclusions are presented in Section 4.

## 2 Character networks

Previously many researchers [32–36] have reported that written human language can be modeled using complex networks. The complexity of Chinese offers several possibilities for defining and studying complex networks. The grammar of modern Chinese derive historically from that ancient Chinese. Ancient and modern Chinese differ somewhat in grammatical rules owing to various social and cultural factors [37]. Therefore, to obtain more information regarding the structural organization and dynamic evolution of Chinese, both ancient and modern Chinese versions of the Records of the Grand Historian (104 B.C.) [38] are considered as research material for our character networks. As noted in the Introduction, we treat both ALN and MLN as weighted directed graphs.

Consequently, we define the networks as follows.

1. Each individual character chosen in the text represents a different network vertex.
2. If two characters are neighbors, then there is an edge between them, with the edge pointing from the former character toward the latter, and the weight w of an edge depends on the frequency (times) of connection between two Chinese characters appearing in the text.
3. The degree of a vertex k is equal to the number of different consequent neighbors that a Chinese character has. In addition, the strength s of a vertex is equal to the number of times (frequency) a Chinese character occurs in a text.

For simplicity, we overlook punctuation in our work and consider the out-degree and out-strength of a vertex as its degree and strength, respectively. This construction results in the different weighted networks ALN and MLN, respectively. We can understand the evolution of written Chinese more fully by studying the two networks.

Some information regarding ALN and MLN can be gathered from the model construction. The network sizes are 957,510 and 505,835, respectively, and the number of different characters is 4,877 and 4,336, respectively. The average intensity of the modern Chinese network is almost 1.7 times as that of the ancient Chinese one. The average degree and weight of MLN are also greater than those of ALN (Table 1). It is natural to wonder why the length of ALN is much less than that of MLN even though the number of vertices of the two articles is nearly the same and whether the connections of Chinese characters play a more important role in modern than in ancient Chinese. We aim to enhance our understanding of weighted language networks through structural analysis of ALN and MLN.

**Table 1:** Some fundamental parameters of the two language networks.

Network	MLN	ALN
**Text length**	957510	505835
**Num.of vertice**	4877	4336
**Num.of edges**	227096	169160
**<k>**	46.56	39.01
**<w>**	4.22	2.99
**<s>**	196.33	116.66

## 3 Analytical results

In Chinese, the most obvious clue to evolution is the dynamic relationship among the characters. Although the two character networks generate in the same manner, some stylistic differences remain with respect to the statistical characteristics of ALN and MLN (Table 1). In other words, these differences might reflect different structural organizations and linguistic rules of ancient and modern Chinese. More information can be collected through analysis of the weighted networks.

In this section, we investigate and discuss some statistical parameters of the two networks. These parameters include Zipf’s law, power law, average nearest-neighbor degree, interaction properties, connectivity, and clustering coefficient. Motivated by these observations, we explore the two character networks.

### 3.1 Zipf ‘s law distribution

Zipf’s law, a consequence of the match between structure and dynamics, states that the frequency of words decays as a power function of its ranks [39, 40]. Actually, several different mechanisms can generate Zipf-like statistics [41]. Recent findings reported a relationship between Zipf’s law and the principle of least effort [39]. Scientists have found that Zipf’s law usually holds in large-scale texts and applies to a variety of languages [34]. Sheng Long [42] discovered that Chinese characters follow a two-part Zipf’s law, with curves that differ from the English ones.

**Fig 1:**
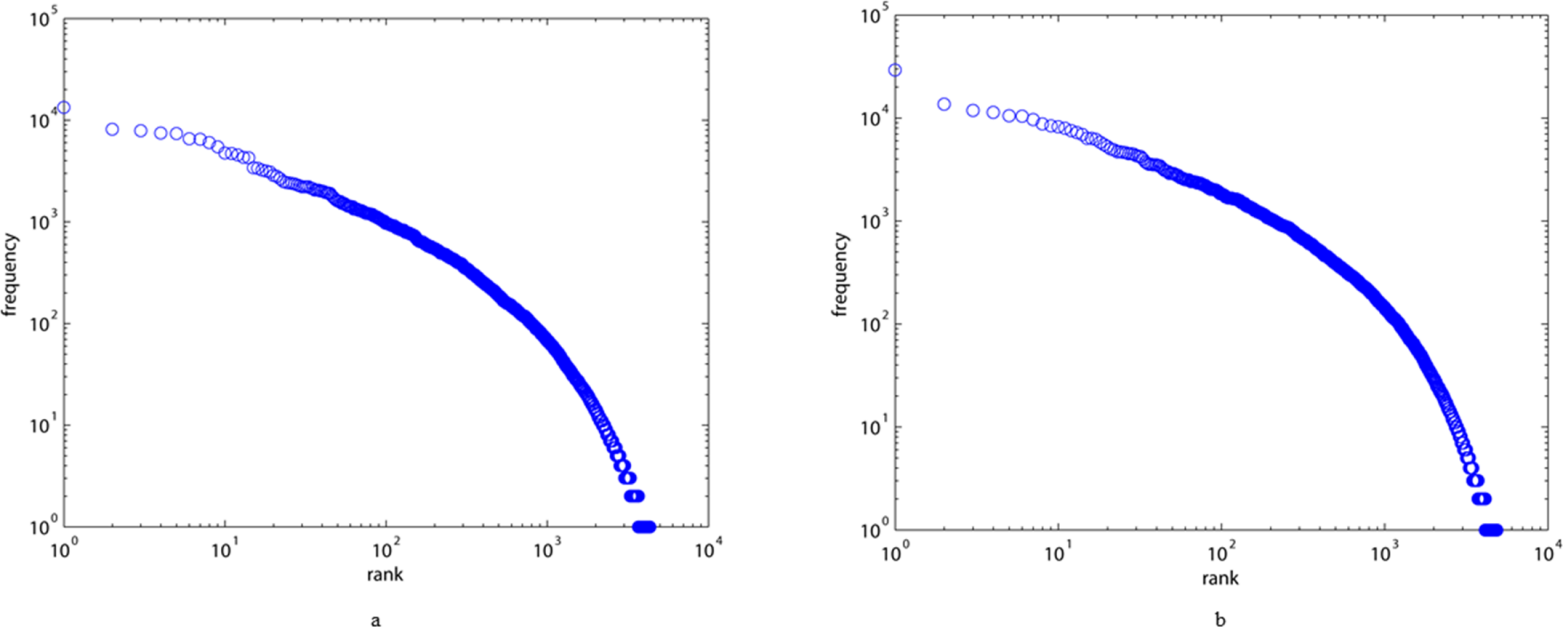
The distribution of Zipf’s law for the ancient Chinese version of Record of the Grand Historian (a) and its modern version (b).

Fig 1 shows measurements of Zipf’s law for ALN and MLN—they all fit the two-part Zipf’s law well. The two-part Zipf’s law of both ALN and MLN might be related to the growth and preferential selection mechanism of characters in Chinese, indicating that Zipf’s law does not depend on the syntactic structure of Chinese.

**Fig 2:**
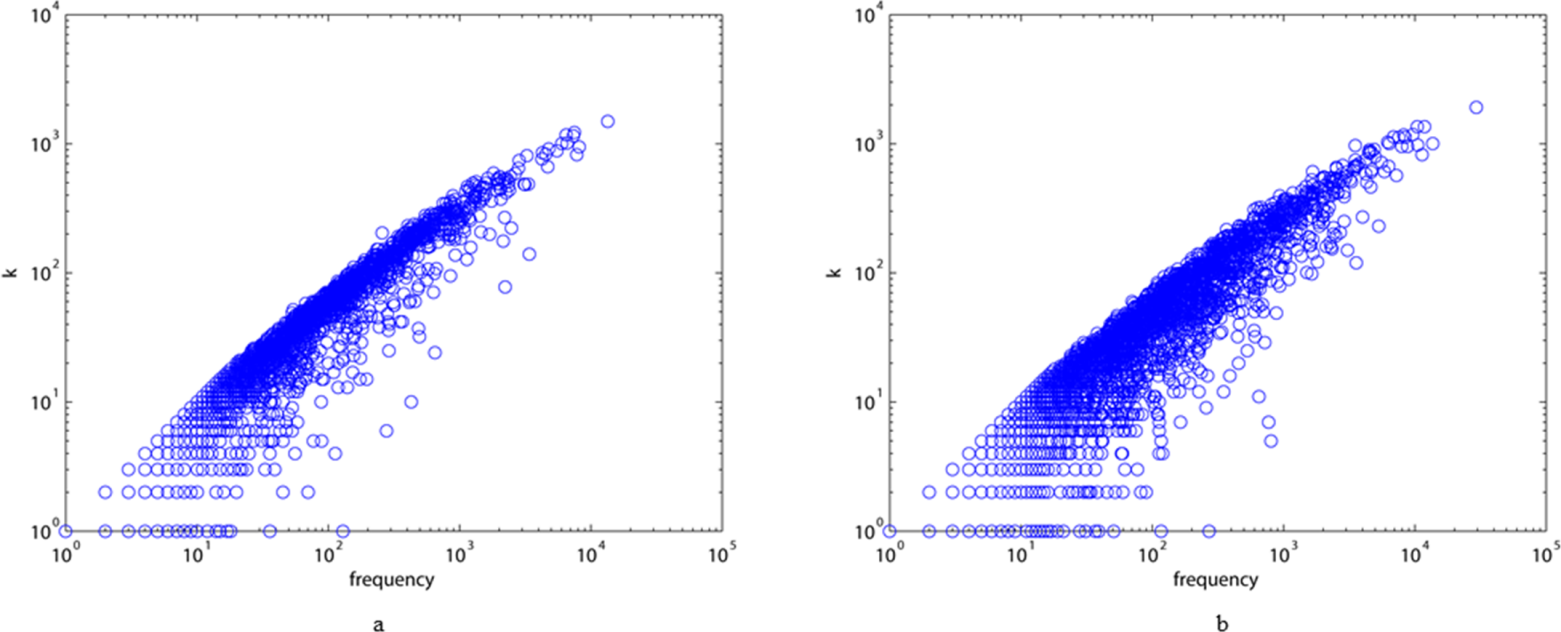
The frequency of characters corresponds to the values of degree k for ALN (a) and MLN (b).

Furthermore, in Fig 2, we show the relationship between vertex frequencies and degrees. As the figure shows, the frequency of a vertex is nearly proportional to its degree in both ALN and MLN, indicating that characters with high degrees also often have high frequencies. Traditionally, the frequency of Chinese characters has been used to judge their importance. For example, over 85,000 Chinese characters have been created historically, but approximately 5,000 are commonly in use, with these being regarded as more important than the others. Consequently, it is necessary to explore the interplay between the frequency and structure of character networks.

### 3.2 Power law distributions of Chinese character networks

In many networks, the power law distribution exhibits scale-free behavior. Thus, the exploration of scale-free properties is important in studying character networks and can be expected to help us comprehend how the language maintains its relative stability in development and evolution. We can conclude that one of our networks is a scale-free network if P(k) satisfies the power law degree distribution

$$P(k)=k^{-\partial }$$ (1)

where P(k) is the probability that a randomly chosen vertex in the network has exactly degree k.

**Fig 3:**
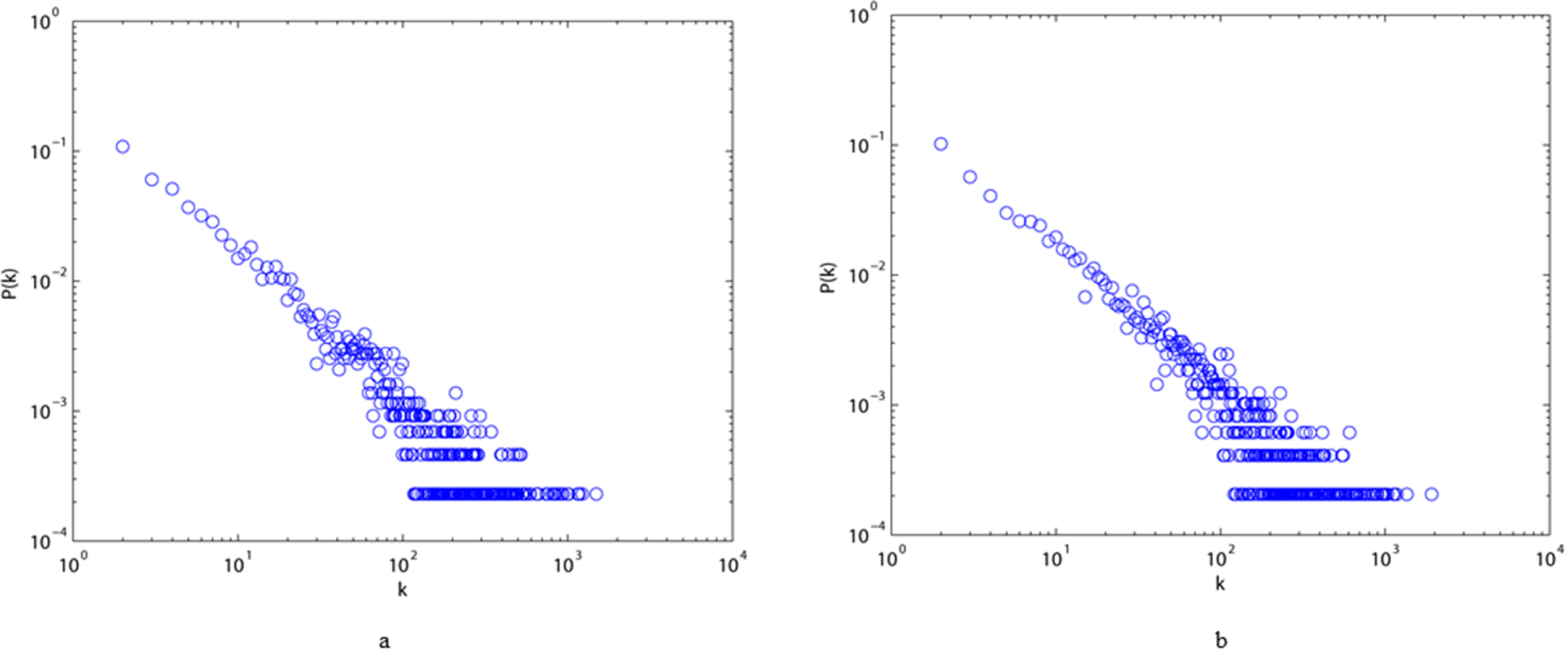
Degree distribution P(k) of ALN (a) and MLN (b) in the log-log scale, which all fit power-law property.

**Fig 4:**
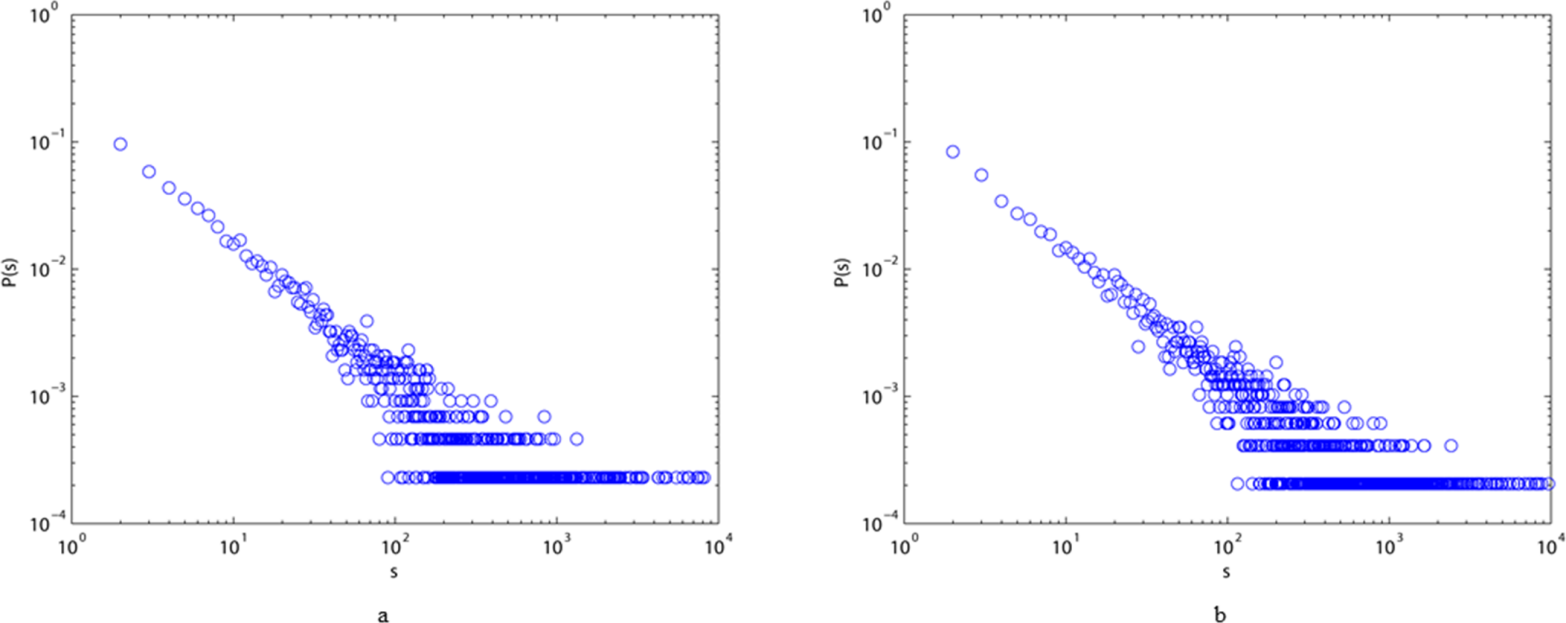
Strength distribution P(s) of ALN (a) and MLN (b) in the log-log scale, which all fit power-law property.

**Fig 5:**
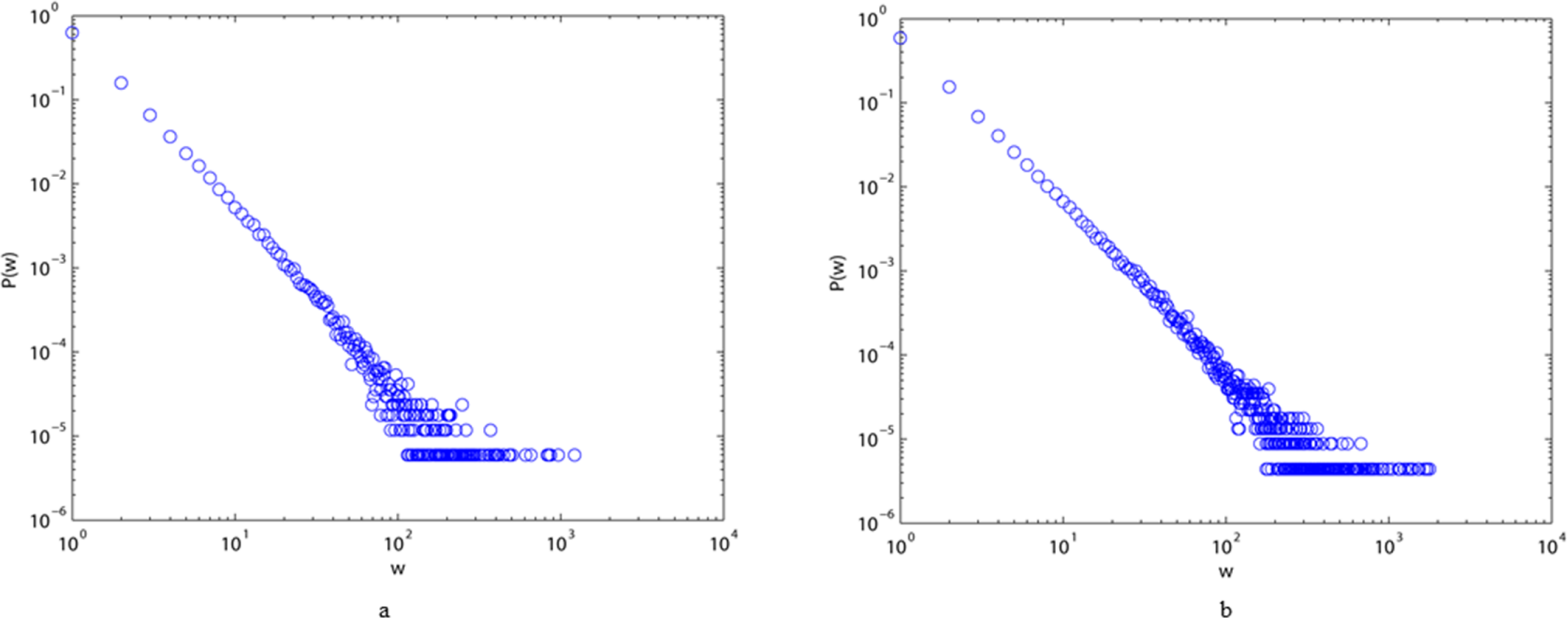
Edge weight distribution P(w) of ALN (a) and MLN (b) in the log-log scale, which all fit power-law property.

We also show typical plots of degree distributions, strength distributions, and distributions of the weights of edges for the two different styles of the language network in Figs 3–5. All these diagrams show downward-sloping lines, indicating that they follow power law distributions. From Fig 3(A) and 3(B), we can conclude that most elements of the two networks are connected to one or two others, and that only a few of them (the hubs) have a very large number of links. Fig 4(A) and 4(B) imply that the majority of vertices in ALN and MLN are of low strength, while some vertices are of high strength. From Fig 5(A) and 5(B), we can see that the majority of the edges in both networks have low weights, while only a few of them have high weights, indicating that the link frequencies among most vertices are small. Thus, we can conclude that most characters have few links to others in both ALN and MLN. Moreover, the power law distribution in Fig 2 can be interpreted as indicating that the two types of Chinese language comprise self-organizing systems, similar to many real-world networks.

### 3.3 Interactive analysis of Chinese character networks

Here, we propose the interaction coefficient q to measure Chinese characters’ capacity for phrase formation. Assuming s and k respectively represent the strength and degree of vertices, the interaction coefficient of a vertex is defined as

q=s/k (2)

In particular, for a phrase consisting of many characters, we expect there to be fixed connections among them. In other words, the strength of a Chinese character increases with the character’s occurrence frequency (f). However, if a character has a low degree (k), then the character and its neighbors are more likely to form phrases. That is to say, we consider characters and their neighbors to have fairly strong interactivity.

**Fig 6:**
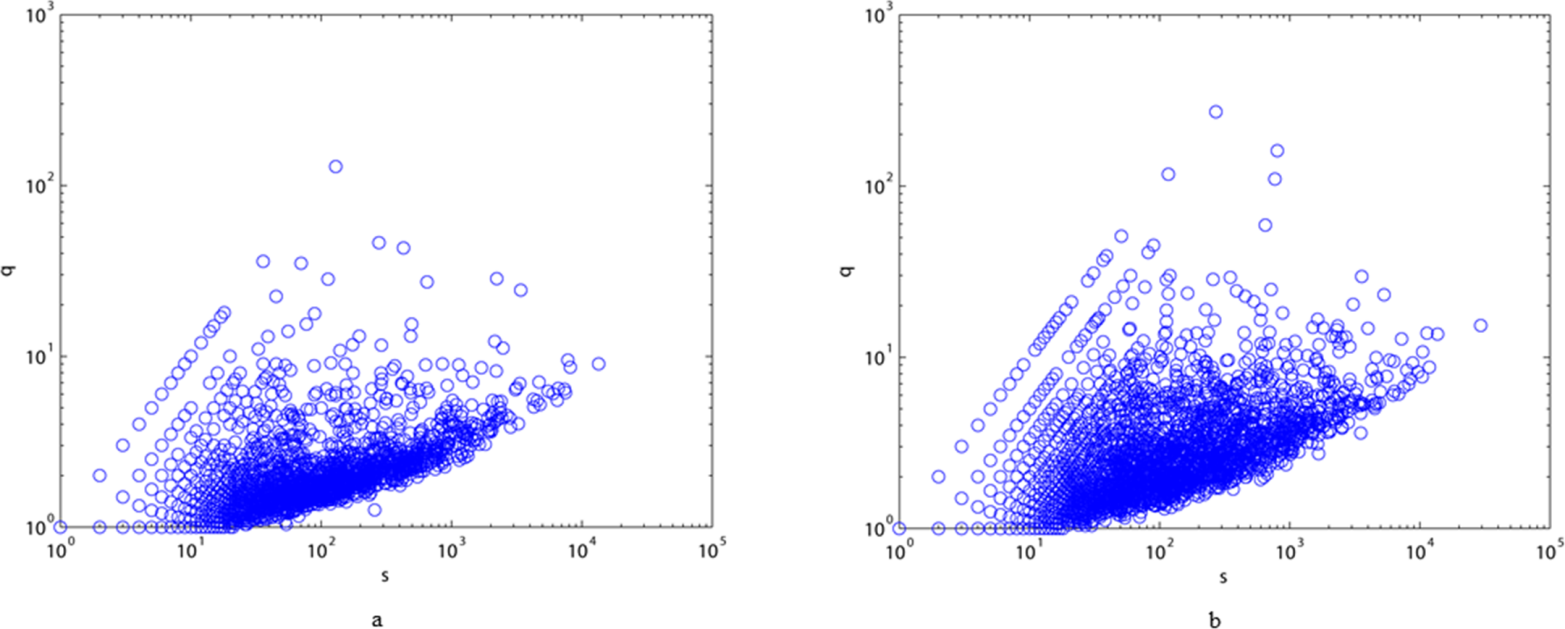
Interaction coefficient q vs. vertex strength s for ALN (a) and MLN (b).

In Fig 6, we show the relationship between the interaction coefficient and vertex strength. Comparing Fig 6(A) and 6(B), we can see that the number of vertices in MLN that have high q values is greater than that in ALN, and that there is a mass of vertices with q below 20, indicating that there are more phrases in modern Chinese than in ancient Chinese. In addition, the interaction coefficient is not an increasing function of strength in each network, indicating that vertices with high strength might simultaneously have high degree, with the result that these vertices do not have high q values. In that case, it is difficult to form phrases in either text, consistent with the phrase forming conditions. In particular, we list the top ten Chinese characters with high degrees and their corresponding strength and interaction coefficients in both ALN and MLN in Table 2. These Chinese characters with high degree and high strength are typically called hubs and are important for both ancient and modern Chinese. We find that these hubs have rather small q values, which can be explained by the fact that people can communicate conveniently by connecting hubs with meaningful characters according to syntax rules. Additionally, the hub changes suggest the presence of stylistic differences between ancient and modern Chinese.

**Table 2:** Chinese characters of top ten degrees and their corresponding strengths and interaction coefficients.

ALN				MLN			
vertex	k	s	q	vertex	k	s	q
**之**	1488	13438	9.03	**的**	1918	29356	15.31
**以**	1227	7502	6.11	**是**	1357	10412	7.67
**而**	1172	6494	5.54	**了**	1355	11823	8.73
**为**	1149	7356	6.40	**人**	1178	9650	8.91
**子**	1011	6584	6.51	**有**	1176	8193	6.97
**曰**	1000	6039	6.04	**在**	1133	6904	6.09
**王**	943	8161	8.65	**子**	1125	8417	7.48
**人**	916	4772	5.21	**为**	1122	7544	6.72
**其**	882	5497	6.23	**到**	1006	6358	6.32
**者**	851	4287	5.04	**国**	999	13638	13.65

### 3.4 Average nearest-neighbor degree

Another important source of information lies in the correlations of the degrees of the neighboring vertices, one of the important indicators in the study of language network evolution [43]. Since the entire conditional distribution P(k*|*k*), a given site with degree k connecting to another site with degree k*, is often difficult to interpret, the average nearest-neighbor degree has been proposed to measure these correlations [44].

$$k_{nn,i}=\frac{1}{k}\sum_{i,j=1}^{N}a_{ij}k_{j}$$ (3)

Once averaged over classes of vertices with degree k, the average nearest-neighbor degree can be defined as

$$k_{nn}(k)=\sum_{k^{*}}k^{*}P(k^{*}|k)$$ (4)

The average nearest-neighbor degree provides a probe for the degree correlation function. If the degrees of the neighboring vertices are uncorrelated, then *k*_*nn*_(*k*) is independent of k and P(k*|*k*), is a function only of k*. An increase in *k*_*nn*_(*k*) with k indicates that high-degree vertices tend to be connected with other high-degree vertices. In such a case, the network is said to be assortative. In contrast, a network is said to be disassortative if *k*_*nn*_(*k*) decreases with increasing k [42]. In fact, the assortative/disassortative properties reflect the evolution of network structure in terms of efficiency and stability.

Analogously, the weighted average nearest-neighbor degree can be expressed [45] as

$$k_{nn}^{w}(k)=\frac{1}{s_{i}}\sum_{i,j=1}^{N}w_{ij}k_{j}$$ (5)

Such quantities are used to characterize the assortative/disassortative properties the behavior of $k_{nn}^{w}(k)$ is an effective measure of the affinity for connecting with high- or low-degree neighbors according to the magnitude of the actual interactions. If $k_{nn}^{w}(k)>k_{nn}(k)$, then edges with larger weights are pointing to neighbors with higher degrees, and $k_{nn}^{w}(k)<k_{nn}(k)$ for the opposite case [46].

To verify global assortativity or disassortativity, we also compute the Pearson correlation coefficient [47], which is within the interval [−1, 1]. If the network is uncorrelated, then r = 0. Assortative networks have a value of r > 0, while disassortative networks have r < 0.

The calculated values of r are −0.2151 and −0.2083 for MLN and ALN, respectively. This indicates that both language networks are disassortative in terms of degree. This disassortative phenomenon in ALN and MLN can be intuitively understood to mean that high-degree vertices are preferentially connected with low-degree ones, resulting in negative correlations with regard to vertex degree. Therefore, characters with relatively high values of degree k tend to form phrases in both texts.

**Fig 7:**
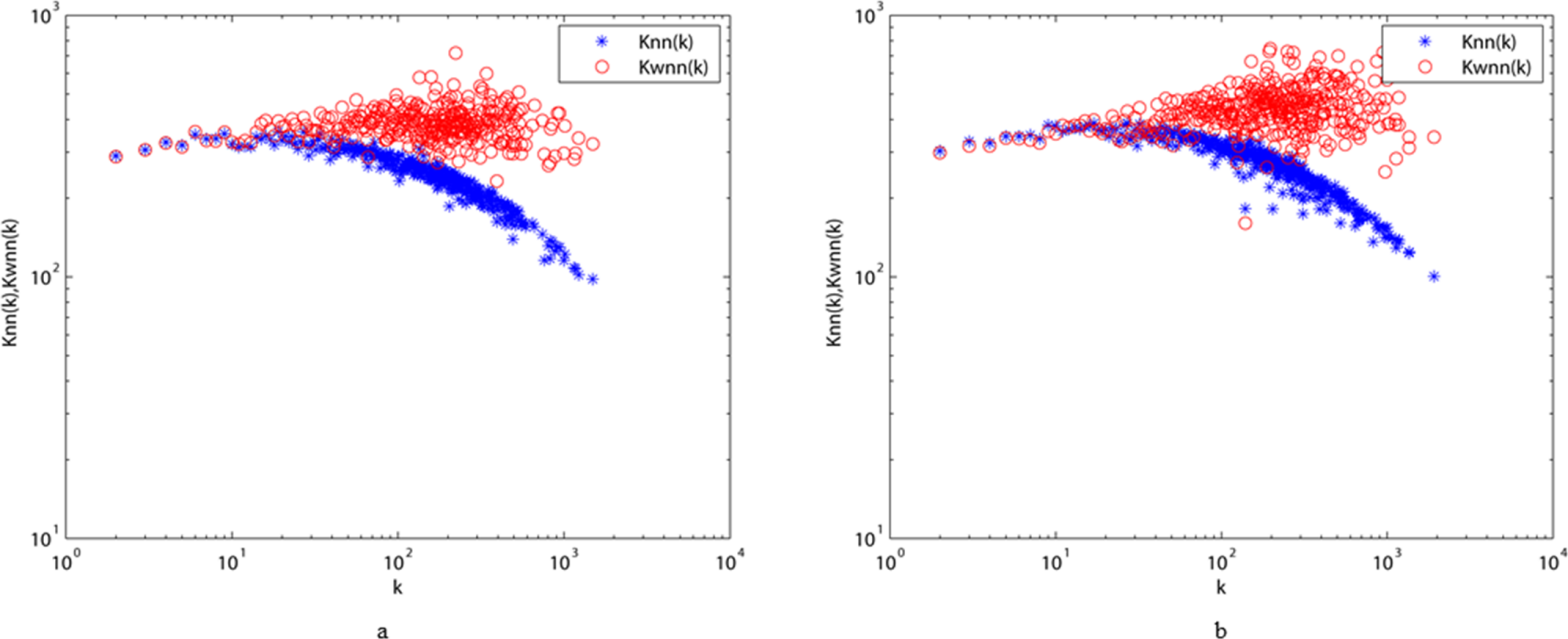
The comparisons of $k_{nn}^{w}(k)$ and *k*_*nn*_(*k*) between ALN (a) and MLN (b) under different values of degree *k*.

Our measurements of $k_{nn}^{w}(k)$, $k_{nn}(k)$ and their comparisons for ALN and MLN are shown in Fig 7. We can see that both ALN and MLN exhibit disassortative properties according to the decreasing curves of *k*_*nn*_(*k*), indicating a nontrivial correlation for the two networks [27]. In addition, $k_{nn}^{w}(k)>k_{nn}(k)$ persists through almost the entire spectrum of degree k in ALN, while $k_{nn}^{w}(k)<k_{nn}(k)$ retains a degree value less than approximately 10 in MLN. This indicates that more edges with large weights appear between hubs and low-degree vertices in MLN than in ALN. While large-weight edges between vertices indicate strong interactions between the characters, this behavior can be interpreted as evidence that MLN displays a greater heterogeneity in character interactions than ALN, which might be another reason for there being more phrases in the modern text than in the ancient one.

### 3.5 Architectural analysis of Chinese character networks

In this subsection, we explore in detail the architectures of the two language networks through weighted network representations of the ancient and modern Chinese versions of the Records of the Grand Historian and find that they exhibit different behaviors.

#### 3.5.1 Clustering coefficient

The first and a widely used measure of complex networks is vertex clustering. The clustering coefficient is considered a measure of the cohesiveness and the quantity of a network’s hierarchical structure [34]. The clustering of a vertex i is defined [42] as

$$c_{i}=\frac{1}{k_{i}(k_{i}-1)}\sum_{j,h\in N}a_{ij}a_{ih}a_{jh}$$ (6)

where *k*_*i*_ is the degree of vertices I and N is the total number of vertices in a network. If vertices i and j have a connection between them, then *a*_*ij*_ = 1; otherwise, *a*_*ij*_ = 0. In addition, further information can be obtained through the average clustering coefficient C(k), defined as

$$C(k)=\frac{1}{NP(k)}\sum_{i/k_{i}=k}c_{i}$$ (7)

In many real networks, the degree-dependent clustering coefficient C(k) is a decreasing function of k, indicating that low-degree vertices generically belong to interconnected groups, while high-degree vertices are linked to many vertices that might belong to different groups that are not directly connected [48,49].

However, Eq (6) does not take into account the fact that in a weighted network, some neighbors are more important than others. Barrat et al. [36] defined the weighted clustering coefficient of a vertex i as

$$c_{i}^{w}=\frac{1}{s_{i}(k_{i}-1)}\sum_{j,h\in N}\frac{\left(w_{ij}+w_{ih}\right)}{2}a_{ij}a_{ih}a_{jh}$$ (8)

where *s*_*i*_ is the strength of vertex i and *w*_*ij*_ is the weight of the edge between vertices i and j. In the general case, the weighted clustering coefficient takes into account both the number of closed triangles in the neighborhood of vertex i and their total relative weight w with respect to strength s.

The average over vertices of a given degree k yields the quantity *C*^*w*^(*k*) [42]. Comparison of *C*(*k*) and *C*^*w*^(*k*) provides global information on the correlation between weights and topology. However, we can face two opposite cases in many real weighted networks. Edges with large weights tend to form triples if *C*^*w*^(*k*) > *C*(*k*), while the opposite situation indicates that the triangles have less relevance [46].

Analyzing the two character networks, we can easily see that the calculated values of the network clustering coefficient C are 0.1243 and 0.1197 for ALN and MLN, respectively. This indicates that fewer characters are required to connect any two characters in ancient Chinese than in modern Chinese. In this case, expressions in ancient Chinese are, in general, briefer than in modern Chinese. Moreover, the high values of C for ALN and MLN indicate that we can deliver or search information in networks more quickly.

**Fig 8:**
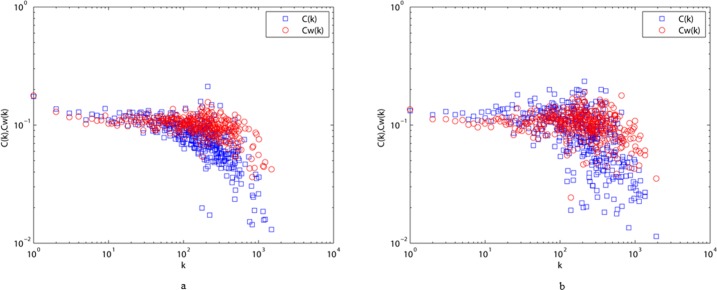
The comparisons of *c*(*k*) and *c*^*w*^(*k*) between ALN (a) and MLN (b) under different values of degree k.

We also compare *C*(*k*) and *C*^*w*^(*k*) for ALN and MLN in Fig 8, revealing some similarities and differences. On one hand, *C*(*k*) and *C*^*w*^(*k*) of the two networks both fluctuate around 0.1 for degree k values less than approximately 500, indicating that ALN and MLN do not entirely display a stronger hierarchical behavior. On the other, *C*^*w*^(*k*) < *C*(*k*) appears for degree k values less than approximately 20 and 80 in ALN and MLN, respectively. This implies that the clustering of low-degree vertices has less influence on the organization of MLN than on that of ALN. Therefore, probing the clustering of ALN and MLN can be expected to help us comprehend the character networks’ architectures, improving our interpretation of the evolution of written Chinese.

### 3.5.2 Connectivity of Chinese character networks

**Fig 9:**
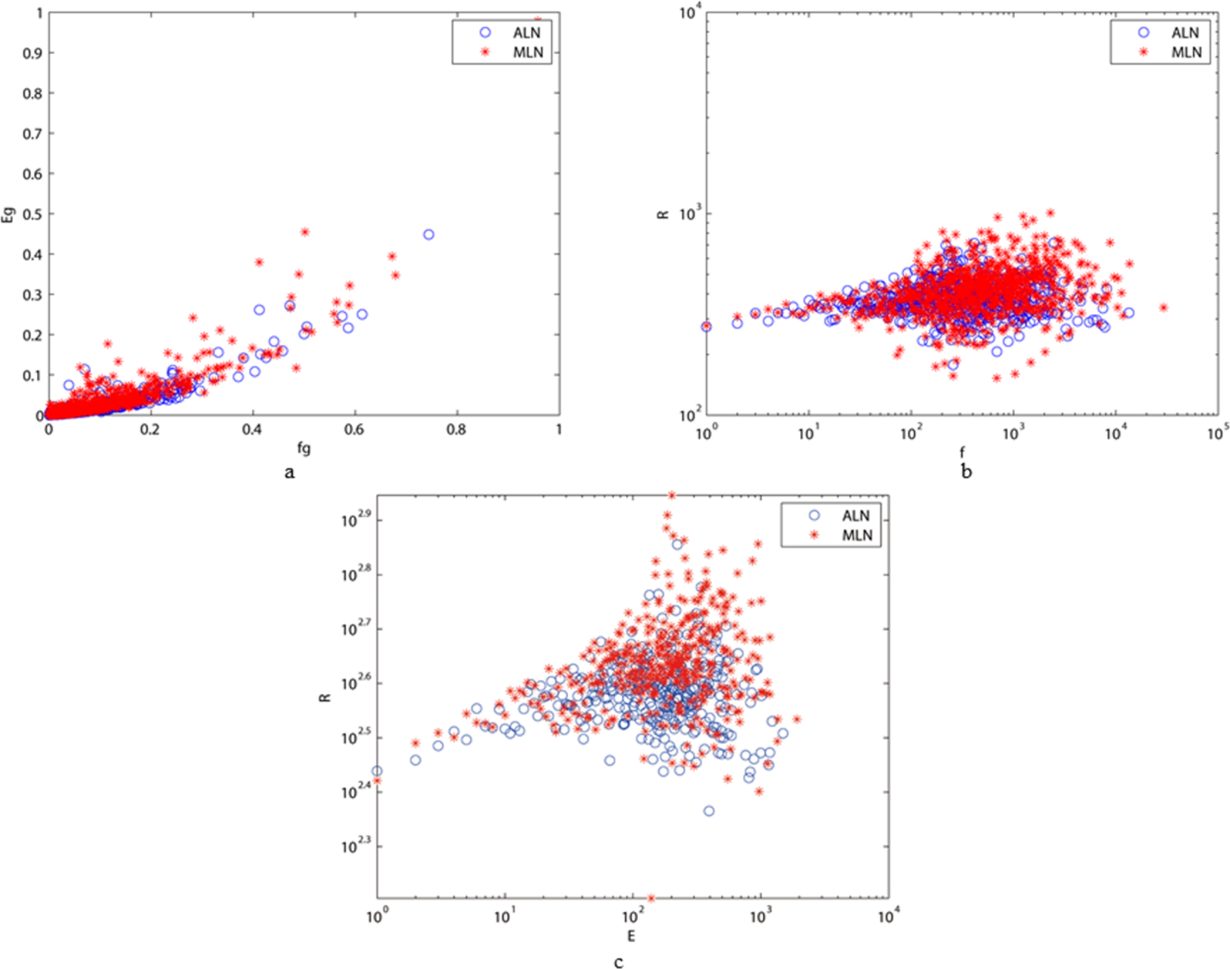
(a) The comparative results of the number of edges E between ALN and MLN restricted to the vertices’ frequency f. The correlations of the degree of neighboring vertices R between ALN and MLN under the vertices’ frequency (b) and the number of edges (c) respectively.

Measuring the connectivity of networks is the fundamental task in network research. Comparison of the connectivities of different networks can reveal the similarities and differences among the networks. Thus, in Fig 9, we compare the characters’ connections, which play an important role in information expression, in ALN and MLN.

Fig 9(A) shows the relationship between the number Eg of edges and frequency fg of vertices. (Eg and fg represent the normalized number of edges and frequency, respectively.) We can see that there are more edges corresponding to high-frequency vertices in MLN than in ALN. Actually, the higher the frequency of occurrence of a vertex, the more corresponding neighbors and edges it has, and it is more likely to form a phrase with its neighbors. Thus, this difference can be attributed to more phrases appearing in the modern Chinese text than in the ancient one.

In Fig 9(B) and 9(C), we also compare the correlations of the degrees of neighboring vertices R restricted to frequency f and the number of edges E, respectively. We can see that the parameters (values of R) of ALN are flatter than those of MLN in both figures, indicating that the structure of MLN varies more with changes in the correlations of the degree of neighboring vertices R compared to ALN. This shows that MLN displays greater heterogeneity in the intensity of character interactions since the parameters of MLN are more dispersive than those of ALN. These facts can be interpreted to provide evidence as to why the length of ALN is much less than that of MLN even though the number of vertices of the two articles is nearly the same. These results indicate that the connections between characters in the modern text have greater intensity and density than those of the ancient one. MLN has greater connectivity than ALN, with the result that phrases occur more in the modern Chinese text than in the ancient one.

## 4 Conclusion

In this study, we analyze in detail the structures of ancient and modern Chinese using weighted network representations of different versions of the same article. We find both differences and similarities in the two forms of Chinese.

First, the ancient Chinese text is much shorter than the modern one even though the number of the different characters of the two texts is nearly the same. In addition, the analysis of the average nearest-neighbor degree implies that both the character networks are disassortative.

Next, through a statistical analysis of the two character networks, we find that both fit the two-part Zipf’s law well and exhibit scale-free behavior. Meanwhile, an interactive analysis of the character networks show that the number of interaction coefficients with high q values in the modern Chinese version is greater than that of in the ancient version, indicating that the modern Chinese text should have more phrases than the ancient one.

Furthermore, a clustering analysis of the two networks reveals that the ancient and modern Chinese language networks do not entirely display a strong hierarchical behavior. The calculation of the network clustering coefficient C indicates that expressions in ancient Chinese are briefer than those in modern Chinese.

Finally, by analyzing the architectures of the two networks, we find that connectivity in the modern Chinese language network is greater than that in the ancient one. This might be a reason why modern Chinese could construct a longer text using fewer characters compared to ancient Chinese.

This study provides a preliminary discussion of Chinese language networks. In our future work, more new and complex models will be considered, which we expect to provide new clues for the formation and evolution mechanisms of other languages.

## Supporting information

S1 Text ALN. (TXT)

S2 Text MLN. (TXT)
